# Prognostic Immune and Nutritional Index as a Predictor of Survival in Patients Undergoing Curative-Intent Resection for Gastric Cancer

**DOI:** 10.3390/medicina61061015

**Published:** 2025-05-29

**Authors:** Soomin An, Wankyu Eo, Sookyung Lee

**Affiliations:** 1 Department of Nursing, Dongyang University, Yeongju 36040, Republic of Korea; 2 College of Medicine, Kyung Hee University, Seoul 05278, Republic of Korea; 3 Department of Clinical Oncology, College of Korean Medicine, Kyung Hee University, Seoul 05278, Republic of Korea

**Keywords:** gastrectomy, monocytes, serum albumin, stomach neoplasm

## Abstract

*Background and Objectives*: The prognostic immune and nutritional index (PINI) was reported to be clinically relevant for colorectal cancer prognosis. Herein, the utility of PINI as a prognostic factor for the survival of patients with gastric cancer (GC) was investigated. *Materials and Methods*: We retrospectively analyzed 492 patients with stage I–III GC, predominantly of Asian descent, who underwent curative-intent gastrectomy. Multivariate Cox regression analysis identified independent predictors of overall survival (OS). Model performance was evaluated using the concordance index (C-index), integrated area under the curve (iAUC), time-dependent AUC, integrated discrimination improvement (IDI), and continuous net reclassification improvement (cNRI). *Results*: The PINI score—calculated as [albumin (g/dL) × 0.9] − [absolute monocyte count (/μL) × 0.0007]—was found to be independently associated with OS (*p* < 0.001). Additional independent prognostic factors included age, body mass index, 5-factor modified frailty index, tumor–node–metastasis (TMN) stage, gastrectomy type, and anemia. The full model that included all significant covariates outperformed the baseline TNM model, showing significantly higher C-index and iAUC values (both *p* < 0.001). Compared with an intermediate model, which excluded PINI, the full model demonstrated a superior C-index and iAUC (both *p* = 0.004). Although the observed improvements in AUC, IDI, and cNRI at 3 years were not statistically significant, the full model achieved significant gains in all three metrics at 5 years, underscoring the added long-term prognostic value of the PINI. *Conclusions*: PINI is a significant independent predictor of survival in patients with GC who underwent curative-intent surgery. Its inclusion in prognostic models enhances the long-term predictive accuracy for survival, supporting its potential role in guiding personalized postoperative management. External validation in larger multi-ethnic prospective cohorts is essential to confirm its generalizability and to establish its role in routine clinical practice.

## 1. Introduction

Gastrectomy is the primary curative treatment for patients with tumor–node–metastasis (TNM) stage I–III gastric cancer (GC); however, these patients are still at risk of recurrence and death. In a recent study by Kanematsu et al., recurrence rates were reported as 2.4% for stage I, 16.9% for stage II, and 44.1% for stage III disease over a median follow-up period of five years. Notably, recurrence risk peaked at 16 months postoperatively in stage II patients and at 11 months in those with stage III disease. The corresponding 5-year relapse-free survival rates were 91.3% for stage I, 79.3% for stage II, and 50.0% for stage III [1]. Consistently, 5-year overall survival (OS) rates vary markedly by disease stage, ranging from approximately 90% for stage I to 68–86% for stage II, and 30–68% for stage III GC [2,3,4].

While the TNM staging system remains the cornerstone for predicting survival in patients with cancer, it does not fully account for biological variability among individuals. Factors such as tumor heterogeneity, differential treatment responses, and patient-specific characteristics can lead to substantial differences in outcomes among patients classified within the same TNM stage [5,6]. Thus, identifying reliable biomarkers to accurately predict survival is still warranted, as they may offer valuable prognostic insights to clinicians, enhance decision-making both before and after surgery, and ultimately improve patient outcomes.

Serum albumin is a well-established prognostic marker in GC, with hypoalbuminemia linked to higher risks of complications and poor survival [7,8]. Nutritional indices incorporating serum albumin—such as the prognostic nutritional index (PNI), geriatric nutritional risk index, and CONUT score—have shown prognostic value [9,10]. Additionally, various combinations with inflammatory or tumor-related markers have also been investigated to enhance predictive accuracy [11,12,13,14,15,16,17,18,19].

Monocyte-based markers have emerged as relevant indicators in cancer progression due to their role in tumor growth, immune evasion, and angiogenesis [20,21,22]. Indices incorporating absolute monocyte count (AMC)—such as the lymphocyte-to-monocyte ratio (LMR), monocyte-to-lymphocyte ratio (MLR), monocyte and lymphocyte count prognostic score, and systemic inflammatory response index (SIRI)—have shown prognostic associations in GC and other malignancies [23,24,25,26]. However, the clinical utility of AMC-based indices remains to be fully validated.

The prognostic immune and nutritional index (PINI), which is calculated as [albumin (g/dL) × 0.9] − [AMC (/μL) × 0.0007], was recently demonstrated to hold potential for predicting survival outcomes in colorectal cancer (CRC) [27]. Subsequent studies validated its predictive utility in patients with stage I–III CRC [28,29].

Although PINI has shown clinical utility in CRC, its relevance and prognostic value in GC remain largely unexplored. No prior studies have systematically evaluated the performance of PINI in patients with stage I–III GC undergoing curative resection. Therefore, this study aims to assess the prognostic significance of the PINI in this population and determine whether its inclusion enhances the predictive accuracy of survival models beyond traditional clinicopathologic factors.

## 2. Materials and Methods

### 2.1. Study Population

This retrospective study included patients who underwent curative-intent gastrectomy for GC at the Kyung Hee University Hospital (Gangdong, Republic of Korea), between October 2006 and July 2018.

Inclusion criteria were as follows: (1) histologically confirmed primary GC based on the Lauren classification [30]; (2) stage I–III disease as defined by the 8th edition of the American Joint Committee on Cancer TNM staging system [31]; and (3) complete (R0) resection with microscopically negative margins.

Exclusion criteria included the following: (1) history of other malignancies within the preceding five years; (2) prior receipt of anticancer therapy; (3) presence of active infection or autoimmune disease; and (4) absence of preoperative blood count or chemistry data.

### 2.2. Baseline Clinical Characteristics

The collected clinicopathological parameters included age, sex, body mass index (BMI), American Society of Anesthesiologists physical status (ASA-PS) classification [32], and the 5-factor modified frailty index (mFI-5) [33,34]. Tumor characteristics included the extent of the primary tumor (T stage), regional lymph node involvement (N stage), TNM stage, and histological type. Additional pathological features included identification of lymphatic, vascular, and perineural invasion. Surgical and treatment-related variables included the type of gastrectomy (TOG), administration of adjuvant therapy, and length of stay (LOS). TOG was dichotomized as total gastrectomy versus partial gastrectomy, with the latter including subtotal, proximal, distal, and pylorus-preserving gastrectomy. Adjuvant chemotherapy was administered to stage II and III patients in accordance with Korean clinical guidelines using either S-1 monotherapy or a combination of capecitabine and oxaliplatin [35]. No patients in this cohort received adjuvant chemoradiation.

Laboratory parameters assessed were serum albumin, white blood cell count (WBC), absolute neutrophil count (ANC), absolute lymphocyte count (ALC), AMC, hemoglobin levels, mean corpuscular volume (MCV), and platelet count.

PINI was calculated using the following formula: [albumin (g/dL) × 0.9] − [AMC (/μL) × 0.0007] [27]. Other albumin- or AMC-based indices included the PNI, MLR, SIRI, and hemoglobin, serum albumin, lymphocyte, and platelet (HALP) score. PNI was determined as 10 × albumin (g/dL) + 0.005 × ALC (/μL) [9]. MLR was calculated as AMC/ALC [24]; SIRI as (ANC × AMC)/ALC [26]; and HALP score as (hemoglobin × albumin × ALC)/platelet count [19].

Additionally, several inflammation-based indices that excluded serum albumin and AMC were evaluated, including the NLR, platelet-to-lymphocyte ratio (PLR), and systemic immune-inflammation index (SII). NLR was calculated as ANC/ALC [23]; PLR as platelet count/ALC [36]; and SII as (platelet count × ANC)/ALC [37].

### 2.3. Statistical Analysis

The OS was defined as the time from the date of gastrectomy to death from any cause. To reduce potential bias and preserve data integrity, continuous variables were analyzed in their original form rather than categorized. These variables are summarized using medians and interquartile ranges (IQRs) to reflect their distribution.

In addition to being analyzed as continuous variables, both age and hemoglobin levels were categorized for specific subgroup analyses. Age was dichotomized at 50 years, which is a threshold commonly used to define early-onset gastric cancer [38]. Hemoglobin levels were classified into anemia and non-anemia according to the following cutoff values for anemia: <12 g/dL for women and <13 g/dL for men [39]. Non-parametric tests, such as the Mann–Whitney U test and the Kruskal–Wallis test, were applied for comparisons of medians between groups, as appropriate.

Kaplan–Meier survival curves were constructed to estimate the 5-year OS and visualize survival probabilities for key prognostic variables, where applicable. Hazard ratios (HRs) and their corresponding 95% confidence intervals (CIs) were calculated using univariate Cox proportional hazards regression. Variables with a *p*-value < 0.05 in univariate analysis were further evaluated in a multivariate Cox regression model. Variables violating the proportional hazards assumption were excluded from multivariate analysis. Multicollinearity was assessed using the variance inflation factor (VIF), and only variables without significant collinearity were retained in the final model. To manage the relatively large number of covariates, a structured stepwise approach was applied. Composite indices (e.g., NLR, PLR, MLR, SII, SIRI, PNI, HALP score, and PINI) and serum albumin were introduced sequentially to avoid overlapping and multicollinearity with their component variables. The final multivariate model was selected based on statistical significance, clinical relevance, and the highest concordance index (C-index) value. For key continuous variables with significant prognostic value, their association with log relative hazard was explored using fractional polynomial modeling to account for potential non-linear effects. This approach allows for the visualization and interpretation of complex, non-linear associations between covariates and HRs—patterns that may not be readily captured through traditional tabular Cox regression outputs.

The discriminatory performance of each model was assessed using the C-index. Statistical comparisons of model performance were conducted using 1000 bootstrap resamples. To evaluate the stability of the predictive model over time, time-dependent C-indices were estimated and plotted across a 10-year follow-up period using bootstrap cross-validation with 1000 repetitions.

Time-dependent area under the curve (AUC) analyses were also conducted to evaluate and compare the predictive accuracy of the models for OS at 3 and 5 years post-surgery using 1000 bootstrap resamples to ensure robust estimation. To provide a comprehensive summary measure of the model discrimination over time, the integrated AUC (iAUC), which quantifies the average AUC across the follow-up period, was computed.

Additionally, integrated discrimination improvement (IDI) and continuous net reclassification improvement (cNRI) were employed to assess the enhancement in OS prediction between models at 3 and 5 years after surgery [40,41,42]. The IDI quantifies the overall improvement in risk prediction offered by a new model, while the cNRI measures how effectively the new model improves the correct classification of patients into higher or lower risk categories compared to the baseline model.

Prognostic nomograms were constructed based on the finalized multivariable models to estimate OS probabilities for individual patients. Each predictor was assigned a score on the “Points” scale at the top of the nomogram according to its prognostic contribution. The total score was calculated by summing the points for all individual variables. The “Total Points” axis at the bottom of the nomogram allowed for summing all variable-specific points and estimating individualized 3-year and 5-year OS probabilities using the corresponding survival probability scales. Internal validation of the nomograms was performed using calibration curves generated from 1000 bootstrap resamples to assess model accuracy and mitigate overfitting. To further strengthen the clinical relevance of our findings, we have included decision curve analysis (DCA) to evaluate the potential clinical utility of the final model in informing treatment decisions. The DCA provides a visual and quantitative assessment of the net benefit of the model across a range of decision thresholds.

Spearman’s rank correlation was used to assess the strength and direction of associations between continuous variables. To further explore non-linear effects, generalized additive models with a gamma distribution were applied, incorporating predictors selected through least absolute shrinkage and selection operator (Lasso) regression.

To evaluate the potential interaction between serum albumin and AMC in predicting OS of patients with GC, we constructed a base model comprising serum albumin and AMC as independent variables. To assess whether the effect of serum albumin levels on OS was modified by AMC levels, we extended this model by adding an interaction term (albumin × AMC), resulting in an extended model. A likelihood ratio test, which evaluates whether the addition of an interaction term significantly improves the model fit, was used to compare the base and extended models. The predictive accuracy and fitting of the model were evaluated by calculating the C-index and the Akaike Information Criterion (AIC). To visualize the relationship between serum albumin levels and OS across different AMC levels, we generated plots of the predicted HRs from the Cox model with the interaction term, fixing AMC at the 25th, 50th, and 75th percentiles. This graphical analysis supported the statistical findings, reinforcing the idea that any interaction between serum albumin and AMC was negligible.

The *p*-values were two-sided, with statistical significance set at *p* < 0.05. All statistical analyses were performed using MedCalc (version 23.0.2) and R software (version 4.4.0).

## 3. Results

### 3.1. Clinical Characterization of the Study Cohort

The study included 492 patients diagnosed with GC, comprising predominantly Asian individuals (96.1%, *n* = 473), with a small proportion of Caucasians (3.9%, *n* = 19). The median age of the participants was 60.5 years (IQR: 52.0–70.0). The TNM stage distribution was as follows: stage I (61.4%, *n* = 302), stage II (18.5%, *n* = 91), and stage III (20.1%, *n* = 99). Concerning the ASA-PS scores, 42 patients had a score of 1, 394 patients had a score of 2, and 56 patients had a score of 3. For the mFI-5 score, 199 patients scored 0, 182 scored 1, 79 scored 2, 26 scored 3, and 6 patients scored 4. Most patients underwent partial gastrectomy (79.1%, *n* = 389), whereas 20.9% (*n* = 103) underwent total gastrectomy (Table 1).

### 3.2. Identification of Risk Factors for OS of Patients with GC

The median follow-up period was 107.5 months (IQR: 58.0–140.9 months). Kaplan–Meier survival analysis showed 5-year OS rates of 92.3%, 76.2%, and 47.4% for patients with cancer at stages I, II, and III, respectively. Univariate Cox regression analysis identified several variables significantly associated with OS (Table 2). In multivariate analysis, when the NLR, PLR, MLR, SII, SIRI, PNI, HALP score, PINI, and serum albumin were introduced sequentially, the model incorporating the PINI yielded the highest C-index of 0.815 (95% CI, 0.784–0.846). The C-indices for models incorporating each of the other markers were as follows: 0.801 (95% CI, 0.768–0.834) for NLR, 0.801 (95% CI, 0.768–0.834) for PLR, 0.806 (95% CI, 0.774–0.838) for MLR, 0.801 (95% CI, 0.768–0.834) for SII, 0.801 (95% CI, 0.768–0.834) for SIRI, 0.801 (95% CI, 0.768–0.834) for HALP score, and 0.810 (95% CI: 0.778–0.841) for serum albumin. These results suggest that the PINI provides superior prognostic discrimination compared to other inflammation- and nutrition-based indices in patients with GC undergoing curative-intent resection.

The finalized multivariate model (full model) included the following significant predictors: age, BMI, mFI-5, TNM stage, TOG, anemia, and PINI. The VIF for the included variables were as follows: 1.06 for age, 1.17 for BMI, 1.20 for mFI5, 1.16 for TNM stage, 1.03 for TOG, 1.29 for anemia, and 1.28 for PINI, indicating negligible multicollinearity among the variables. All the significant variables adhered to the proportional hazards assumption (Table 2).

**Figure 1:**
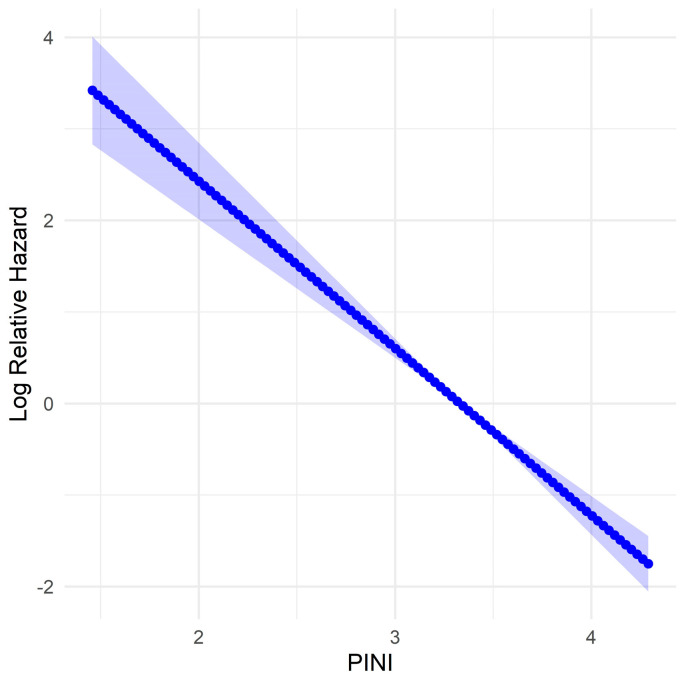
Fractional polynomial model of log relative hazard for the prognostic immune and nutritional index (PINI) for overall survival (OS) in patients with gastric cancer (GC). The shaded area represents the 95% confidence interval.

Fractional polynomial analysis showed that higher PINI scores were associated with a lower relative risk of mortality, as indicated by the consistent decline in log relative hazard (Figure 1).

When stratified by adjuvant therapy status, the prognostic value of the PINI score for predicting OS remained significant in both subgroups. The adjusted HR for PINI was 0.30 (95% CI: 0.16–0.56; *p* < 0.001) in patients who did not receive adjuvant therapy and 0.35 (95% CI: 0.22–0.57; *p* < 0.001) in those who did, supporting PINI as a robust and independent prognostic indicator of OS, regardless of adjuvant treatment status.

Among patients evaluated for recurrence within 36 months after surgery, those without recurrence had a significantly higher median PINI score (3.41; IQR: 3.37–3.46) compared to those with recurrence (3.08; IQR: 2.97–3.26; *p* < 0.001). When stratified by recurrence pattern, median PINI scores were 3.17 for locoregional, 3.17 for peritoneal, and 3.05 for distant recurrence. Statistically significant differences were observed between the non-recurrence group and both the peritoneal (*p* = 0.002) and distant recurrence groups (*p* < 0.001). Similarly, among patients who received adjuvant chemotherapy, recurrence-free individuals also had a significantly higher median PINI score (3.35; 95% CI: 3.32–3.44) compared to those who experienced recurrence (3.03; 95% CI: 2.93–3.16; *p* < 0.001). Within this subgroup, median PINI scores were 3.16 for locoregional, 3.06 for peritoneal, and 3.02 for distant recurrence, with significant differences again observed between the non-recurrence group and both the peritoneal (*p* = 0.007) and distant (*p* < 0.001) recurrence groups. These findings highlight the prognostic utility of preoperative PINI in identifying patients at increased risk of early and systemic recurrence, even among those receiving adjuvant therapy.

### 3.3. Comparison of Different OS Predictive Models

The full model, which included all significant variables identified in the Cox regression analysis (age, BMI, mFI-5, TNM stage, TOG, anemia, and PINI), exhibited enhanced predictive accuracy compared with both the baseline (based solely on TNM stage) and intermediate (incorporated all variables from the full model except for the PINI) models. Compared with the baseline model, the full model demonstrated a significantly higher C-index for OS (0.815 vs. 0.659, *p* < 0.001). Similarly, the full model achieved substantially higher iAUC values for OS (0.791 vs. 0.640, *p* < 0.001), indicating an improved predictive performance over time. In addition, the full model achieved substantially higher AUC values for 3- and 5-year OS (all *p* < 0.001), indicating an enhanced discrimination. IDI and cNRI analyses also confirmed significant improvements for postoperatively predictive accuracy and risk stratification with the full model at both 3 and 5 years (all *p* < 0.001).

Compared with the intermediate model, the full model achieved a notably higher C-index (0.815 vs. 0.797, *p* = 0.004), highlighting the added prognostic value of including PINI for survival prediction. Similarly, the full model achieved substantially higher iAUC values for OS (0.791 vs. 0.776, *p* = 0.004), indicating improved predictive performance over time. For the 3-year OS, the full model demonstrated higher AUC, IDI, and cNRI values; however, these differences were not statistically significant. Regarding the 5-year OS, the full model exhibited statistically significant improvements in AUC (*p* = 0.012), IDI (*p* = 0.032), and cNRI (*p* = 0.012), indicating the added long-term prognostic value of the PINI. Collectively, these findings underscore the enhanced predictive capacity of the full model, particularly during long-term follow-up (Table 3).

**Figure 2:**
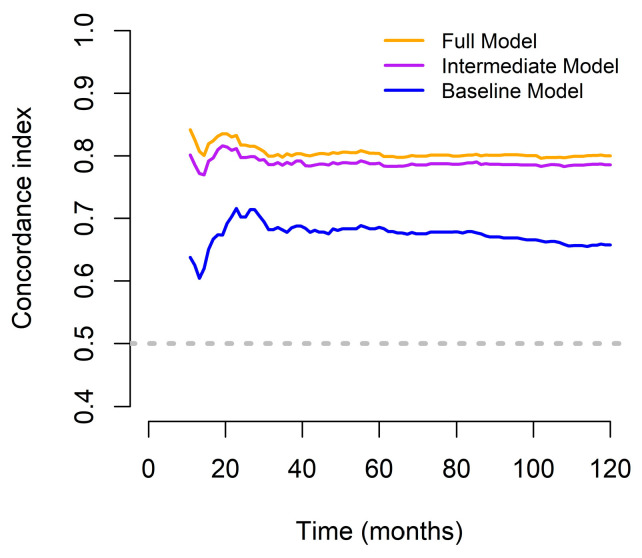
Concordance indices of the predictive models designed to assess OS outcomes in patients with GC for over a 10-year period.

The predictive advantage of the full model was consistently maintained over the 10-year follow-up period, as illustrated by the time-dependent concordance index plot (Figure 2), which represents the discriminatory ability of each model to predict OS at different time points. The full model consistently achieved the highest C-index across the entire follow-up period, maintaining stable and superior predictive accuracy (approximately 0.80–0.82). Excluding PINI (intermediate model) led to a slightly lower, but still robust discrimination performance. In contrast, the baseline model, which included only the TNM stage, showed substantially lower C-index values (approximately 0.65–0.70), indicating limited predictive capability. Notably, although all three models performed significantly better than chance, the sustained advantage of the full model highlights the additive value of including comprehensive clinical, functional, and nutritional parameters, particularly the PINI. These results confirm that the full model offers a more accurate and consistent survival prediction across long-term follow-up, supporting its clinical utility in postoperative risk stratification (Figure 2).

### 3.4. Establishment and Validation of the Constructed Nomogram

**Figure 3:**
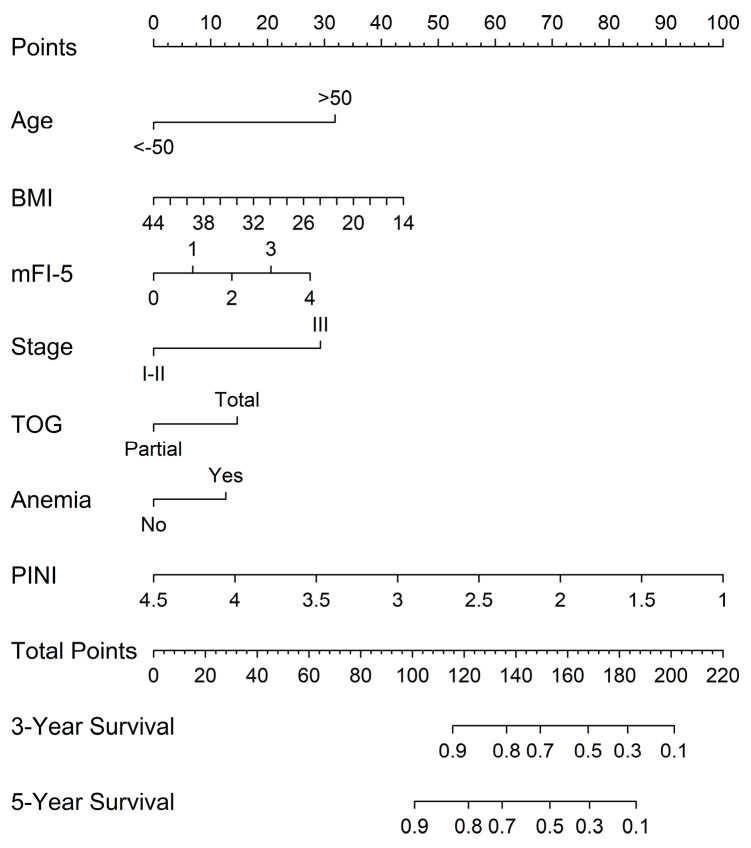
Nomograms predicting 3-year and 5-year OS based on the full model.

A prognostic nomogram for OS in patients with GC was developed using the full model. Overall, older age, lower BMI, higher mFI-5 score, advanced stage (stage III), total gastrectomy, presence of anemia, and lower PINI scores contributed to a higher total score, reflecting a worse prognosis (Figure 3).

**Figure 4:**
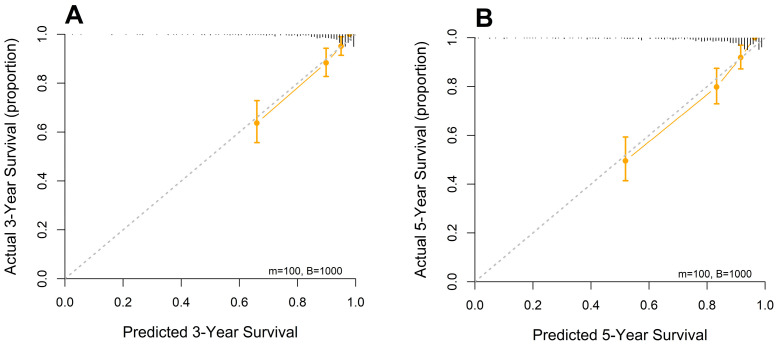
Calibration curves of the designed nomogram for predicting (**A**) 3- and (**B**) 5-year OS.

Calibration plots were generated for 3-year and 5-year OS probabilities, showing excellent concordance between the predicted and observed survival outcomes, with a particularly strong alignment in patients with higher predicted survival probabilities (Figure 4).

**Figure 5:**
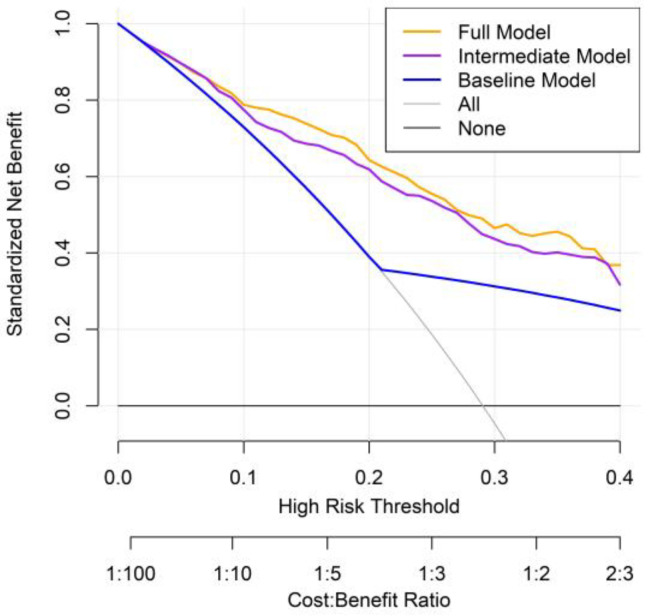
Decision curve analysis comparing prognostic models for predicting overall mortality in patients with GC.

DCA was conducted to evaluate the clinical utility of three prognostic models: the baseline model, the intermediate model (excluding PINI), and the full model (including PINI) (Figure 5). Across a range of clinically relevant threshold probabilities (0–40%), the full model consistently demonstrated the highest net benefit, followed by the intermediate model and then the baseline model. This pattern suggests that incorporating PINI into the predictive model provides incremental clinical value in identifying high-risk patients who may benefit from closer surveillance or intervention. Notably, the full model offered superior net benefit, particularly in the threshold range of 10% to 30%, where clinical decisions about perioperative management are most likely to be considered.

### 3.5. Evaluation of Predictors for PINI

Spearman’s correlation analysis was performed across multiple clinical and laboratory parameters. The PINI demonstrated a strong positive correlation with serum albumin (*r* = 0.94) and PNI (*r* = 0.71), indicating a substantial overlap in the nutritional and inflammatory information captured by these indices. In contrast, the PINI exhibited only weak correlations (∣*r*∣ < 0.5) with the remaining variables, suggesting that it provides distinct prognostic information relative to other common clinical markers.

To explore the determinants of PINI, Lasso regression analysis was conducted using a comprehensive set of clinical, pathological, and laboratory variables. Candidate predictors included categorical variables such as age group (≤50 vs. >50 years), sex, ASA-PS, mFI-5, T stage, N stage, overall TNM stage, histologic type, lymphatic, vascular, and perineural invasion, type of gastrectomy (total vs. partial), receipt of adjuvant therapy (yes vs. no), anemia status (based on sex-specific hemoglobin thresholds), and tumor location. Additionally, continuous variables from routine blood tests and nutritional markers were incorporated to capture a broad range of potential influences on PINI.

Using LASSO for variable selection, the final model retained serum albumin (coefficient = 0.8804) and AMC (coefficient = −0.00065) as the only predictors of PINI. The model demonstrated excellent performance, with an *R*^2^ value of 0.9992 and a root mean square error of 0.0112 on the test set, indicating an exceptionally strong fit. These findings suggest that serum albumin and AMC are the primary determinants of PINI expression.

To further refine the model, a generalized additive model with gamma distribution was fitted (intercept at 1.196; *p* < 0.001), confirming the significance of both serum albumin and AMC. The smooth terms for s(albumin) and s(AMC) had 8.744 and 6.083 estimated degrees of freedom, respectively, within the predefined basis dimensions (*k* = 9), supporting adequate model flexibility. Both predictors remained highly significant (*p* < 0.001) and the model showed excellent calibration, with an adjusted *R*^2^ of 0.999 and 99.8% deviance. Residual diagnostics confirmed the appropriateness of the gamma distribution, with no evidence of model misspecification. In summary, the PINI demonstrated a strong correlation with its constituent components—serum albumin and AMC—while showing no substantial associations with demographic factors, tumor pathology, or other clinical parameters, highlighting its specificity as a composite inflammatory and nutritional biomarker.

### 3.6. Comparison of the Prognostic Discriminatory Ability of the PINI Score vs. Serum Albumin and AMC

Based on the iAUC analysis, the PINI demonstrated a slightly better discriminatory ability than the serum albumin (iAUC: 0.699 vs. 0.691), although the difference was not statistically significant (*p* = 0.142). In contrast, PINI showed significantly superior performance compared with AMC (iAUC: 0.699 vs. 0.564, *p* < 0.001). These results suggest that, while PINI and serum albumin perform similarly, PINI offers a clear advantage over AMC in predicting patient outcomes.

**Figure 6:**
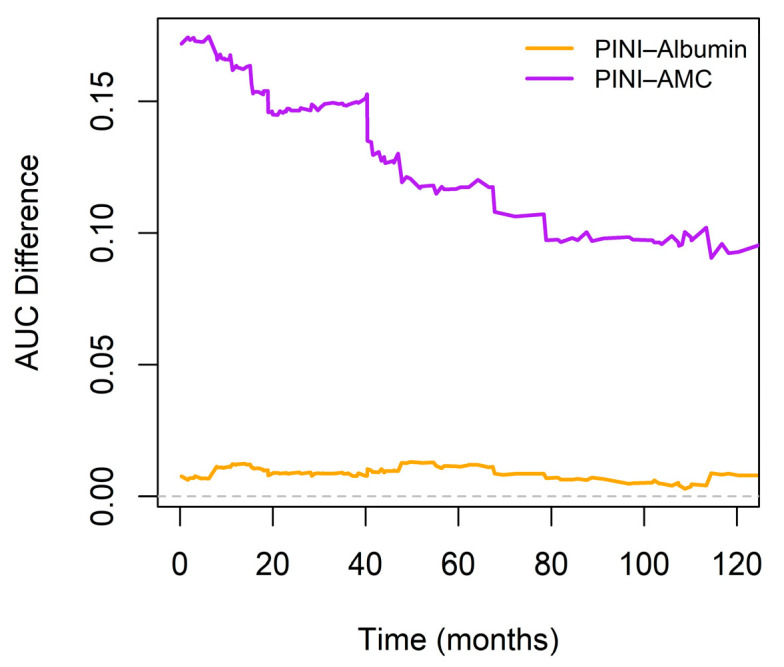
Time-dependent differences in area under the curve (AUC) between PINI and serum albumin or absolute monocyte count (AMC). The horizontal dashed line at 0 marks the reference.

Pointwise time-dependent differences in the AUC between the PINI and serum albumin over a 120-month follow-up period further showed a slight, yet sustained, improvement in the discriminatory ability of PINI (orange line). In contrast, the AUC difference between PINI and AMC (purple line) was substantially higher, particularly within the first 60 months, and remained favorable thereafter (Figure 6).

To further evaluate the predictive performance of PINI, we developed the albumin-substituted model (ASM) by substituting the PINI with serum albumin. Of note, BMI was excluded from this model because of its lack of statistical significance in the multivariate analysis. Thus, the significant variables in the ASM were age (HR, 0.32; *p* = 0.004), mFI-5 (HR, 1.26; *p* = 0.005), TNM stage (HR, 2.96; *p* < 0.001), TOG (HR, 1.60; *p* = 0.010), anemia (HR, 1.79; *p* = 0.002), and serum albumin (HR, 0.38; *p* < 0.001). No significant multicollinearity was observed between the variables.

When comparing model discrimination, the full model demonstrated a slightly higher C-index than the ASM (0.815 vs. 0.810, *p* = 0.280), suggesting a modest improvement in predictive accuracy. A similar result was observed when comparing model discrimination, with the full model demonstrating a slightly higher iAUC than the ASM (0.791 vs. 0.787; *p* = 0.180) (Table 4).

### 3.7. Evaluation of Possible Interaction Between Serum Albumin and AMC

To evaluate the potential interaction between serum albumin and AMC in predicting OS in patients with GC, we constructed two Cox proportional hazard models. The base model included serum albumin and AMC as independent variables, whereas the extended model included an interaction term (albumin × AMC). We compared these nested models using the likelihood ratio test, which yielded a non-significant result (*p* = 0.990), indicating that the interaction term did not enhance the explanatory power of the model. Consequently, a simpler model (without the interaction term) was deemed sufficient to describe the relationship among serum albumin, AMC, and OS in this GC cohort. The performance metrics consistently showed that the base model had a slightly better C-index (0.4665 vs. 0.4629) and a lower AIC (1577.4 vs. 1579.4) than the interaction model. Overall, the interaction between the two variables was statistically negligible.

**Figure 7:**
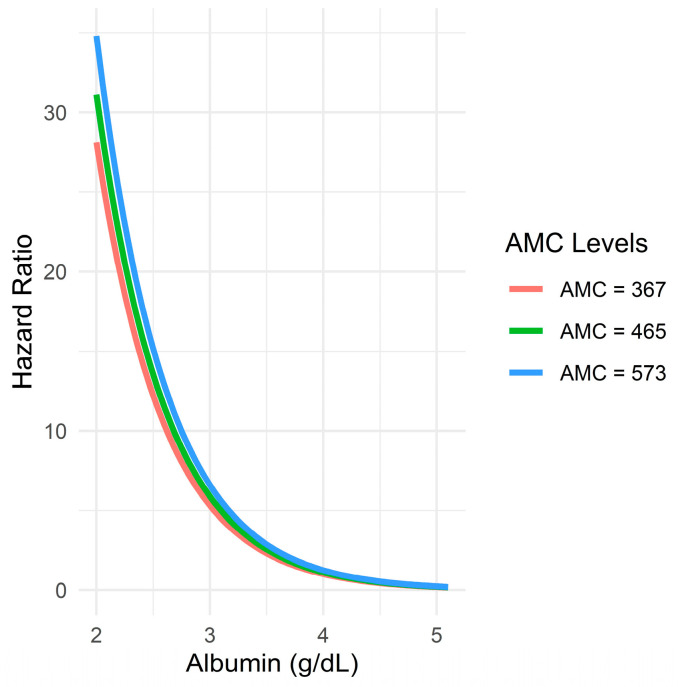
Relationship between serum albumin levels and predicted hazard ratio across different AMC levels.

The predicted HRs from a Cox proportional hazards model that included an interaction term between serum albumin and AMC (fixed at the 25th, 50th, and 75th percentiles further revealed that higher serum albumin levels were associated with significantly lower HRs regardless of the AMC value. The slope was the steepest at lower albumin levels, indicating a disproportionately high risk in patients with hypoalbuminemia. Moreover, no meaningful divergence in the albumin–hazard relationship was observed across the different AMC strata, as denoted by the nearly superimposed curves. This supports the earlier interaction test result (*p* = 0.990), reinforcing that any statistical interaction between serum albumin and AMC was negligible or nonexistent (Figure 7).

## 4. Discussion

In our study, the PINI emerged as an important prognostic factor for OS in patients with GC who underwent curative-intent gastric resection. The PINI stands out as a robust tool for prognostic assessment owing to its simplicity and reliance on two fundamental components, serum albumin and peripheral AMC count, both of which are readily available without additional expenses. Moreover, our comprehensive full model, which integrates the PINI with other significant variables, demonstrated superior predictive accuracy compared with conventional models, such as those based solely on the TNM stage. Since the PINI encompasses the critical aspects of immune function and nutritional status, it offers a holistic approach to prognostic evaluation with more nuanced risk stratification for patients with GC.

PINI was strongly associated with serum albumin and AMC, but not with other demographic or pathological variables, indicating its independence. Albumin reflects nutritional reserves and systemic inflammation and has been widely validated as a prognostic factor in GC [7,8]. Numerous composite indices incorporating serum albumin have been linked to survival outcomes, further emphasizing its clinical relevance [9,10,11,12,13,14,15,16,17,18,19]. Inflammatory cytokines like TNF-α and IL-6 can suppress albumin synthesis, contributing to poorer prognosis [7,8,43,44,45,46]. AMC has also emerged as a marker of tumor-promoting immune activity, and its inclusion in PINI underscores the index’s relevance to cancer biology [20,21,22].

In our multivariate analysis, age, BMI, mFI-5, TNM stage, TOG, and anemia were identified as independent predictors of OS in GC. Patients with late-onset GC (>50 years) had significantly poorer outcomes, consistent with findings by He et al., who attributed this to greater comorbidity and cumulative exposure to carcinogens [38]. Low BMI was also associated with worse survival, aligning with Wada et al.’s report that underweight patients had inferior OS compared to those with normal or high BMI [47]. TNM stage remained a strong, independent predictor of prognosis, reaffirming its central role in GC staging and survival prediction [5,48,49,50,51,52]. Total gastrectomy was linked to poorer outcomes, likely due to its higher risk of postoperative nutritional complications and morbidity [51,53,54]. Additionally, preoperative anemia emerged as a significant factor; a meta-analysis of over 13,000 GC patients found that 36% were anemic and had significantly reduced survival (HR = 1.33) [55].

We initially included NLR, PLR, MLR, SII, SIRI, PNI, HALP score, and serum albumin when constructing the comprehensive predictive model. However, these variables were excluded from the final model due to their relatively lower statistical significance in multivariate analysis. The variability in findings across studies may be attributed to differences in methodological approaches, such as the use of categorical vs. continuous variable analyses, which can affect the detection of prognostic associations. Additionally, the heterogeneity in patient cohorts, including demographic and clinical characteristics, may influence the prognostic value of these markers. Therefore, although we did not identify these markers as independent prognostic factors within our specific cohort or analytical framework, their potential utility in other contexts remains valid and warrants further investigation.

The full model, which included age, BMI, mFI-5, TNM stage, TOG, anemia, and PINI, outperformed both the baseline and intermediate models in predicting OS. Specifically, when compared with the baseline model (relying solely on the TNM stage), the full model yielded a significantly higher concordance index (*p* < 0.001) and improved iAUC (*p* < 0.001), reflecting enhanced discriminatory ability across the follow-up period. In addition, the full model showed higher AUCs for 3- and 5-year OS (*p* < 0.001 for both). The IDI and cNRI analyses also confirmed improved risk discrimination and reclassification at both time points (*p* < 0.001), and the calibration curves showed good agreement between the predicted and observed outcomes. Moreover, the full model achieved a higher C-index (*p* = 0.004) and iAUC (*p* = 0.004) as compared with the intermediate model. While differences in the 3-year AUC, IDI, and cNRI were not statistically significant, the full model showed significant improvements in all three metrics for 5-year OS (*p* = 0.012, 0.032, and 0.012, respectively), highlighting the added prognostic value of PINI over the long-term follow-up. Taken together, these results confirm that the full model offers enhanced discrimination, calibration, and clinical utility in predicting long-term survival.

The predictive advantage of the full model was consistently maintained throughout the 10-year follow-up period, as shown in the time-dependent C-index plot. The full model demonstrated the highest concordance index across all time points, indicating stable and superior discriminatory ability for OS. The intermediate model, which excluded the PINI, showed a slightly lower but still acceptable performance, whereas the baseline model, based solely on the TNM stage, had notably lower C-indices, reflecting limited predictive power. All models performed above the chance level; however, the sustained advantage of the full model underscores the value of integrating clinical, functional, and nutritional factors, particularly PINI, for accurate long-term survival prediction and postoperative risk stratification.

In this study, we evaluated whether there was a statistically and clinically meaningful interaction between the components of the PINI (serum albumin and AMC) in predicting OS among patients with GC. The base model included serum albumin and AMC as independent variables, whereas the extended model included an interaction term (albumin × AMC). Model performance metrics, including the C-index and AIC, consistently indicated that the base model either outperformed or matched the extended model, suggesting that the interaction between albumin and AMC was negligible. Moreover, the prognostic impact of serum albumin remained stable across varying AMC levels, reinforcing its independent predictive value.

Although serum albumin was confirmed as a strong and independent prognostic factor, the PINI demonstrated a slightly higher iAUC than serum albumin alone, although these differences were not statistically significant. Moreover, over the entire 10-year follow-up period, the PINI consistently showed superior discriminative performance in terms of AUC relative to serum albumin, suggesting a potential incremental benefit in long-term prognostic assessment. A comparison of the models also revealed that the full model incorporating PINI achieved marginally higher C-index and iAUC values than the ASM, further suggesting a modest enhancement in predictive accuracy. These findings imply that, while albumin captures a substantial portion of the prognostic signal, PINI may offer incremental value by incorporating inflammatory information via AMC. By encompassing a broader spectrum of nutritional and inflammatory statuses, the PINI provides a new platform for personalized treatment for patients with GC.

Given its simplicity and ability to capture both nutritional and inflammatory status, the PINI shows strong potential as a practical and accessible tool for preoperative risk stratification in patients undergoing curative-intent gastrectomy for GC. In our study, the full model incorporating PINI consistently identified patients at higher risk of adverse outcomes. This model may help clinicians tailor perioperative strategies such as nutritional support, prehabilitation, and enhanced postoperative monitoring for patients who are not otherwise classified as high risk based on traditional clinicopathologic factors. The nomogram developed from the full model provides a user-friendly visual tool for individual risk prediction and could be incorporated into clinical workflows or decision support systems. Notably, DCA demonstrated that the full model offered superior net clinical benefit, particularly within the 10–30% threshold range—a zone in which treatment decisions are often nuanced and individualized. This reinforces the utility of PINI in guiding perioperative care and identifying patients who may benefit from closer follow-up or additional interventions.

Furthermore, subgroup analyses provided additional insight into the potential role of PINI in guiding multimodal management. When stratified by adjuvant therapy status, the prognostic value of PINI for OS remained significant in both treated and untreated subgroups, underscoring its robustness as an independent predictor. Among patients evaluated for recurrence within 36 months postoperatively, those without recurrence had significantly higher preoperative PINI scores than those who relapsed, particularly in cases of peritoneal or distant metastasis. This pattern persisted even among patients who received adjuvant chemotherapy, suggesting that lower PINI values are associated with early and systemic recurrence despite adjuvant therapy. These findings highlight the utility of preoperative PINI in identifying high-risk patients who may benefit from intensified surveillance, closer follow-up, or adjunctive perioperative interventions.

To confirm the generalizability of PINI, future studies should aim to validate the full model incorporating PINI in large, multiethnic, and prospectively collected cohorts. Particularly valuable would be its use in clinical trials as a stratification variable or risk-adjustment factor in evaluating perioperative interventions, such as nutritional support programs or immune-nutrition protocols. Prospective studies should also examine the utility of serial PINI measurements—before and after surgery—to assess whether dynamic changes in systemic inflammation and nutritional status predict recurrence or treatment response. This temporal assessment could further enhance PINI’s clinical relevance and guide individualized follow-up intensity or timing.

In addition, while biomarkers such as human epidermal growth factor receptor 2, microsatellite instability, and Epstein–Barr virus status are increasingly recognized for their relevance in GC biology and treatment, their prognostic significance in patients undergoing curative resection remains limited [56,57,58]. Integrating these molecular features with systemic inflammatory and nutritional markers like PINI may enable more robust and personalized prognostic tools that reflect the biological heterogeneity of GC. Ultimately, incorporating PINI into clinical decision-support algorithms, predictive nomograms, and trial designs may support more refined, multimodal, and risk-adapted management strategies for patients with GC.

This study had several strengths. To the best of our knowledge, this is the first study to evaluate the prognostic significance of PINI in patients with GC undergoing curative-intent resection. The PINI, derived from readily available and cost-effective parameters, such as serum albumin and AMC, serves as a simple yet powerful tool for risk stratification. By treating PINI as a continuous variable, we were able to capture more detailed information, strengthen the statistical analysis, and improve the model’s ability to apply to different patient groups. This approach also revealed a clear, direct relationship between PINI and mortality risk—something that might have been missed if PINI had been grouped into categories. Importantly, we conducted a thorough evaluation of the potential interaction between serum albumin and AMC for predicting OS, which revealed no statistically significant interaction effect and that the prognostic impact of serum albumin was consistent across varying AMC levels. The comprehensive model integrating PINI with clinical and functional parameters demonstrated superior predictive performance across multiple statistical metrics, including the C-index, iAUC, AUC, IDI, and cNRI, particularly for long-term survival prediction. These findings were further corroborated by a time-dependent C-index analysis. Building upon these results, we developed a nomogram incorporating PINI along with other significant clinical variables to predict the 3- and 5-year OS probabilities for GC patients after curative resection. The nomogram exhibited accurate calibration and reasonable discrimination, offering a practical tool for individualized prognostic assessment and aiding clinicians in tailoring postoperative management strategies.

Despite its strengths, this study had several limitations. First, its retrospective design introduces the potential for selection bias and residual confounding, as not all clinical or biological variables can be fully accounted for. Second, the study was conducted at a single center in East Asia, with a predominantly Korean patient population (96.1%). While this ensures internal consistency and data quality, it limits the external validity and generalizability of the findings to broader, multiethnic populations. Differences in genetic background, environmental exposures, dietary habits, and healthcare access may influence the prognostic utility of PINI. Therefore, caution is warranted when applying these findings to non-Korean populations, and external validation in multinational, heterogeneous cohorts is essential. Recent studies have highlighted the importance of such validation across diverse populations [59,60,61]. Third, the predominance of early-stage disease (61.4% stage I) may limit the applicability of our results to patients with advanced or borderline resectable GC, where preoperative biomarkers may have greater clinical relevance. Fourth, although the sample size was adequate for model development, validation in larger, multicenter cohorts is needed to confirm reproducibility and clinical utility. Fifth, a key limitation of this study is the reliance on a single preoperative measurement of the PINI score. Nutritional and inflammatory statuses are inherently dynamic and may vary throughout the perioperative and adjuvant treatment periods. As such, a one-time measurement may not fully capture the evolving risk profile of patients. While our findings support the prognostic utility of baseline PINI for risk stratification, future studies should investigate the value of serial PINI assessments to determine whether temporal changes correlate with recurrence, treatment response, or survival outcomes. Such dynamic monitoring may enhance the clinical applicability of PINI in tailoring postoperative care. However, it should be noted that adjuvant chemotherapy could influence PINI scores, and the type and timing of therapy may further complicate its interpretation—posing a methodological challenge for future longitudinal studies. Finally, as with all observational studies, causal relationships cannot be established and unmeasured confounders may have affected the results. Future prospective studies involving diverse populations are needed to validate and refine the role of PINI in personalized prognostication of GC.

## 5. Conclusions

Our study demonstrated that PINI is a simple, low-cost, and readily available index that can help predict overall survival in patients with gastric cancer undergoing curative-intent resection. By capturing both nutritional and inflammatory status, PINI shows promise as a tool for risk stratification in clinical practice. Clinicians may consider incorporating PINI into predictive models to identify high-risk patients who may benefit from closer monitoring or additional supportive care. Future research should aim to validate PINI in more diverse populations and investigate its potential role in guiding individualized treatment strategies.

## Figures and Tables

**Table 1:** Baseline characteristics of study population with gastric cancer (GC).

Variables	*n* (%) or Median (IQR)	Variables	*n* (%) or Median (IQR)
Age, years	60.5 (52.0–70.0)	Tumor size, cm	3.0 (2.0–5.5)
Sex		Lymphatic invasion	
Men	330 (67.1)	No	325 (66.1)
Women	162 (32.9)	Yes	167 (33.9)
ASA-PS		Vascular invasion	
I/II	436 (88.6)	No	466 (94.7)
III	56 (11.4)	Yes	26 (5.3)
mFI-5 score		Perineural invasion	
0–1	381 (77.4)	No	442 (89.8)
2–4	111 (22.6)	Yes	50 (10.2)
BMI, kg/m^2^	23.7 (21.4–26.0)	Length of stay, days	9 (8–11)
Location		Adjuvant therapy	
Upper	49 (10.0)	No	320 (65.0)
Middle	168 (34.1)	Yes	172 (35.0)
Lower	267 (54.3)	WBC, per μL	6500 (5345–7850)
Diffuse	8 (1.6)	ANC, per μL	3660.5 (2899.5–4806.0)
T stage		ALC, per μL	1907.5 (1534.5–2326.0)
0–1	334 (67.9)	AMC, per μL	465.0 (367.0–573.5)
2–4	158 (32.1)	Hemoglobin, g/dL	13.1 (11.3–14.2)
N stage		MCV, fL	92.2 (88.5–95.5)
0	320 (65.0)	Platelet, ×10^3^ per μL	238.0 (204.5–281.0)
1–3	172 (35.0)	NLR	1.9 (1.4–2.7)
TNM stage		PLR	122.8 (96.7–159.1)
I–II	393 (79.9)	MLR	2.3 (1.8–3.1)
III	99 (20.1)	SII	459.4 (303.8–682.7)
Histology		SIRI	0.9 (0.6–1.3)
Intestinal	243 (49.4)	Albumin, g/dL	4.1 (3.9–4.3)
Others	249 (50.6)	PNI	51.2 (47.2–54.7)
Type of gastrectomy		HALP score	44.5 (30.4–58.5)
Partial	389 (79.1)	PINI	3.4 (3.2–3.6)
Total	103 (20.9)		

**Table 2:** Cox regression analysis for overall survival (OS) of patients with GC.

Covariate	Univariate HR (95% CI)	*p*-Value	Multivariate HR (95% CI)	*p*-Value
Age, years (≤50 vs. >50)	0.16 (0.08–0.35)	<0.001	0.32 (0.15–0.69)	0.004
Sex (female vs. male)	0.59 (0.40–0.87)	0.008		
BMI, kg/m^2^	0.91 (0.87–0.96)	<0.001	0.95 (0.90–1.00)	0.044
ASA-PS score ^†^	2.04 (1.42–2.91)	<0.001		
mFI-5 ^†^	1.50 (1.28–1.76)	<0.001	1.28 (1.08–1.52)	0.004
TNM stage (IIIA vs. I/II)	4.51 (3.23–6.31)	<0.001	2.87 (1.99–4.13)	<0.001
Histology (intestinal vs. others)	1.05 (0.75–1.45)	0.787		
Lymphatic invasion (yes vs. no)	2.79 (2.01–3.89)	<0.001		
Vascular invasion (yes vs. no)	3.32 (1.97–5.59)	<0.001		
Perineural invasion (yes vs. no)	1.99 (1.25–3.17)	0.004		
Tumor size	1.18 (1.14–1.22)	<0.001		
TOG (total vs. partial)	2.35 (1.66–3.33)	<0.001	1.70 (1.19–2.42)	0.003
LOS, days	1.02 (1.01–1.04)	<0.001		
Adjuvant therapy (yes vs. no)	2.48 (1.78–3.45)	<0.001		
WBC	1.00 (1.00–1.00)	0.829		
AMC	1.00 (1.00–1.00)	0.003		
Anemia (yes vs. no)	3.57 (2.55–5.01)	<0.001	1.58 (1.07–2.32)	0.020
MCV	0.97 (0.95–0.99)	0.008		
Platelet	1.00 (1.00–1.00)	0.126		
Albumin	0.18 (0.14–0.25)	<0.001		
NLR	1.16 (1.09–1.23)	<0.001		
PLR	1.01 (1.00–1.09)	<0.001		
MLR	1.42 (1.29–1.57)	<0.001		
SII	1.00 (1.00–1.00)	<0.001		
SIRI	1.21 (1.11–1.32)	<0.001		
PNI	0.87 (0.85–0.90)	<0.001		
HALP score	0.97 (0.96–0.98)	<0.001		
PINI	0.16 (0.11–0.22)	<0.001	0.36 (0.25–0.52)	<0.001

^†^ Ordinal variable.

**Table 3:** Model comparisons for predicting OS in patients with GC.

Metrics	Full Model (FM)	Intermediate Model (IM)	Baseline Model (BM)	FM vs. BM Difference (SE)	*p*-Value	FM vs. IM Difference (SE)	*p*-Value
C-index	0.815 (0.017)	0.797 (0.017)	0.659 (0.020)	0.159 (0.018)	<0.001	0.018 (0.007)	0.004
iAUC	0.791 (0.015)	0.776 (0.017)	0.640 (0.019)	0.144 (0.019)	<0.001	0.014 (0.006)	0.004
3-year OS							
AUC	0.835 (0.024)	0.821 (0.026)	0.690 (0.032)	0.144 (0.027)	<0.001	0.013 (0.011)	0.156
IDI				0.106 (0.027)	<0.001	0.018 (0.016)	0.206
cNRI				0.394 (0.066)	<0.001	0.134 (0.072)	0.054
5-year OS							
AUC	0.857 (0.020)	0.839 (0.022)	0.711 (0.027)	0.146 (0.022)	<0.001	0.019 (0.009)	0.012
IDI				0.141 (0.029)	<0.001	0.031 (0.016)	0.032
cNRI				0.383 (0.054)	<0.001	0.171 (0.062)	0.012

**Table 4:** Model comparisons for predicting survival outcomes in patients with GC.

Metrics	Full Model (FM)	Albumin-Substituted Model (ASM)	FM vs. ASM Difference (SE)	*p*-Value
C-index	0.815 (0.017)	0.810 (0.025)	0.005 (0.005)	0.280
iAUC	0.791 (0.015)	0.787 (0.015)	0.003 (0.004)	0.180
AUC				
3-year OS	0.835 (0.024)	0.833 (0.025)	0.002 (0.007)	0.872
5-year OS	0.857 (0.020)	0.851 (0.021)	0.006 (0.006)	0.412

## Data Availability

The data sets presented in this study are available upon request from the corresponding author due to ethical reasons.

## Abbreviations

The following abbreviations are used in this manuscript:

AIC	Akaike Information Criterion
ALC	Absolute lymphocyte count
AMC	Absolute monocyte count
ANC	Absolute neutrophil count
ASA-PS	American Society of Anesthesiologists physical status
ASM	Albumin-substituted model
AUC	Area under the curve
BMI	Body mass index
C-index	Concordance index
CI	Confidence interval
cNRI	Continuous net reclassification improvement
DCA	Decision curve analysis
GC	Gastric cancer
HALP	Hemoglobin, albumin, lymphocyte, and platelet
HR	Hazard ratio
iAUC	Integrated AUC
IDI	Integrated discrimination improvement
IQR	Interquartile range
Lasso	Least absolute shrinkage and selection operator
LMR	Lymphocyte-to-monocyte ratio
LOS	Length of stay
MCV	Mean corpuscular volume
mFI-5	Five-factor modified frail index
MLR	Monocyte-to-lymphocyte ratio
NLR	Neutrophil-to-lymphocyte ratio
OS	Overall survival
PINI	Prognostic immune and nutritional index
PLR	Platelet-to-lymphocyte ratio
PNI	Prognostic nutritional index
SII	Systemic immune-inflammation index
SIRI	Systemic inflammation response index
TAM	Tumor-associated macrophage
TNM	Tumor–node–metastasis
TOG	Type of gastrectomy
VIF	Variance inflation factor
WBC	White blood cell
